# Microbial Growth Study on Pork Loins as Influenced by the Application of Different Antimicrobials

**DOI:** 10.3390/foods10050968

**Published:** 2021-04-28

**Authors:** David A. Vargas, Markus F. Miller, Dale R. Woerner, Alejandro Echeverry

**Affiliations:** Department of Animal and Food Sciences, Texas Tech University, Lubbock, TX 79409, USA; andres.vargas@ttu.edu (D.A.V.); mfmrraider@aol.com (M.F.M.); dale.woerner@ttu.edu (D.R.W.)

**Keywords:** indicator bacteria, chlorine dioxide, rhamnolipids, 1,3-Dibromo-5.5-dimethyl hydantoin, interventions

## Abstract

The use of antimicrobials in the pork industry is critical in order to ensure food safety and, at the same time, extend shelf life. The objective of the study was to determine the impact of antimicrobials on indicator bacteria on pork loins under long, dark, refrigerated storage conditions. Fresh boneless pork loins (*n* = 36) were split in five sections and treated with antimicrobials: Water (WAT), Bovibrom 225 ppm (BB225), Bovibrom 500 ppm (BB500), Fit Fresh 3 ppm (FF3), or Washing Solution 750 ppm (WS750). Sections were stored for 1, 14, 28, and 42 days at 2–4 °C. Mesophilic and psychrotrophic aerobic bacteria (APC-M, APC-P), lactic acid bacteria (LAB-M), coliforms, and *Escherichia coli* were enumerated before intervention, after intervention, and at each storage time. All bacterial enumeration data were converted into log10 for statistical analysis, and the Kruskal–Wallis test was used to find statistical differences (*p* < 0.05). Initial counts did not differ between treatments, while, after treatment interventions, treatment WS750 did not effectively reduce counts for APC-M, APC-P, and coliforms (*p* < 0.01). BB500, FF3, and WS750 performed better at inhibiting the growth of indicator bacteria when compared with water until 14 days of dark storage.

## 1. Introduction

In 2017, the United States produced almost 52 billion pounds of red meat, of which 25.6 billion pounds of the total were pork [1]. The United States Department of Agriculture (USDA) data show that, in 2018, the per capita consumption of pork was close to 50.8 pounds per year [2]. Pork has always been one of the major meat sources for people, so it is crucial for the industry to ensure a safe pork supply [3].

The Center for Disease Control and Prevention (CDC), in 2018, estimated that one out of six Americans get sick, and from those who were sick, 128,000 were hospitalized, and 3000 died of foodborne diseases [4]. Moreover, the contribution of meat to foodborne illnesses caused by bacteria is 23.20% (beef: 13.20%, pork: 9.80%, and game: 0.10%) [4]. Although the contribution of pork in foodborne illnesses caused by bacteria is lower when compared with beef, it remains significant.

The Institute of Food Science and Technology defines shelf life as “*the period of time during which the food product will remain safe; be certain to retain its desired sensory, chemical, physical, microbiological, and functional characteristics; where appropriate, comply with any label declaration of nutrition data, when stored under the recommended conditions*” [5]. In order to determine shelf life of products, there are series of different methods and equipment that can be used in relation with sensory characteristics of a product, such as color, odor, structure, and flavor, and how these attributes change with time. These types of equipment have been developed to obtain an objective measurement at the moment of analyzing sensorial characteristics of a product.

Furthermore, consumers expect that foods are free of foodborne pathogens and have a decently long shelf life, where antimicrobials play a substantial role in order to achieve this demand [6]. Food antimicrobials are classified as preservatives, according to the U.S. Food and Drug Administration, which are any chemicals that, when added to food, tend to prevent or retard deterioration [6]. The most common function of an antimicrobial is to prolong shelf life through the process of killing or inhibiting spoilage microorganisms while maintaining and extending all the organoleptic properties [6]. It is important to consider that antimicrobials are never a substitute for good sanitation practices in food processing plants, since low initial counts will always be ideal. Although antimicrobials extend the lag phase, their effects on the surviving population can be overcome through time [6]. The global economy in which we live leads us to store and transport food and assure that the food arrives in the condition that is expected, and this is where antimicrobials undoubtedly play a role.

The purpose of this study was to determine the impact of selected antimicrobial spray products on the microbial growth of indicator bacteria naturally present on pork loins after long term storage under dark and refrigerated conditions.

## 2. Materials and Methods

### 2.1. Sample Collection

The study was repeated three times between January to August of 2019. On each repetition, vacuum packaged boneless pork loins (*n* = 36) were purchased from a commercial pork processing plant located in Oklahoma and transported within five hours in a cooler covered with ice at 0–4 °C to the Gordon W. Davis Texas Tech University Meat Science Laboratory (Lubbock, Texas, TX, USA). Pork loins were stored under dark conditions (no light) at 0–4 °C and processed 24 h later.

### 2.2. Treatment Preparation

Treatments were prepared two to three hours before application to the boneless pork loins. For each treatment, three liters of solution was prepared and then stored in a handheld sprayer (Chapin 1-Gallon Plastic Tank Sprayer, Chapin, Batavia, NY, USA). Treatments utilized included: cold water, Bovibrom 225 ppm (1,3-Dibromo-5,5-dimenthylhydantoin; prepared in a mixer provided by Passport Food Safety Solutions, West Des Moines, IA, USA), Bovibrom 500 ppm (prepared the same as Bovibrom 225 ppm), Fit Fresh 3 ppm (chlorine dioxide; prepared following label instructions, Selective Micro Technologies, Dublin, OH, USA), and Natural Washing Solution 750 ppm (rhamnolipid, Jeneil Biosurfactant, Saukville, WI, USA).

### 2.3. Treatment Application

Pork loins were split into five sections of 8.90 cm in length. Each section was randomly assigned to one of the five treatments. For each treatment, 12 pork loin sections were obtained (*n* = 180). Interventions were sprayed onto the pork loin sections for 30 s using a handheld sprayer (Chapin 1-Gallon Plastic Tank Sprayer, Chapin, Batavia, NY, USA; Flow rate: 5.98 ± 0.75 mL/s). Then, sections were flipped and sprayed for another 30 s, ensuring coverage of the entire loin surface. After 10 min, treated sections were vacuum packaged using Cryovac bags (Sealed Air, Charlotte, NC, USA) and randomly assigned to one of the four dark storages periods (1, 14, 28, and 42 days) and refrigerated at temperatures ranging between 0 and 4 °C.

### 2.4. Swab Sample Collection

Buffer peptone water (BPW) pre-hydrated 25 mL swabs (3M, St. Paul, MN, USA), were taken at multiple periods of time during pork processing: before application of intervention, after application of intervention (10 min after finishing interventions), and at the end of each storage time (immediately after opening the bag). For swabs in sections, a 100 cm^2^ template was used. The swabs were taken from the fat and the lean portions of the pork loin sections.

### 2.5. Swabs Sample Processing

After arrival to the laboratory, pre-hydrated swabs were homogenized for two minutes at 230 rpm using an automated stomacher (Steward Laboratory Systems, Davie, FL, USA), serial dilutions with BPW were conducted and plated in Petrifilm (3M, St. Paul, MN, USA) or plates (Thermo Fisher Scientific, Waltham, MA, USA) according to each microorganism.

### 2.6. Total Aerobic Plate Counts

For Aerobic Plate Counts, the Association of Official Agricultural Chemists 990.12 (AOAC) official method was used. After serial dilutions were performed, Petrifilms were placed on a flat surface, inoculated with 1 mL of sample dilution following product instructions. Petrifilms were left undisturbed for one minute to permit gel to solidify. Petrifilms were incubated for 48 ± 3 h at 35 ± 1 °C for mesophilic bacteria conditions and 72 ± 3 h at 20 ± 1 °C for psychrotrophic bacteria conditions. Enumeration was conducted using 3M Petrifilm Plate Reader (3M, St. Paul, MN, USA) and checked in a standard colony counter following the rules of the official method [7,8,9,10,11].

### 2.7. Coliforms and Escherichia coli Enumeration

For Coliforms and *Escherichia coli*, the AOAC 991.14 official method was used. After serial dilutions were performed, Petrifilms were placed on a flat surface. Then one mL of sample was inoculated onto the center of the film base and covered with the top film in duplicates. Petrifilms were left undisturbed for one minute to permit gel to solidify. Petrifilms were incubated for 48 ± 3 h at 35 ± 1 °C. Enumeration was conducted at 24 h for coliforms and 48 h for *Escherichia coli* in a standard colony counter following rules of the official method [12,13].

### 2.8. Lactic Acid Bacteria Enumeration

After serial dilutions, one mL of sample was inoculated on a petri dish and pour plated with 20 mL of Mann–Rogosa–Sharpe Agar (MRS) in duplicates. Plates were placed in BD GasPak EZ Container Systems (Becton Dickinson and Company, Franklin Lakes, NJ, USA) and incubated at 48 ± 3 h at 35 ± 1 °C under microaerophilic conditions (6 to 16% O_2_ and 2 to 10% CO_2_) using BD GasPak EZ Campy Sachets (Becton Dickinson and Company, Franklin Lakes, NJ, USA), [14,15]. Enumeration was conducted using a Q-Counter (Spiral Biotech Inc, Norwood, MA, USA)

### 2.9. Statistical Analysis

Pork loin section swabs before and after interventions was a 2 × 2 factorial design (Sampling point × Treatment) with two levels under sampling point (before and after) and 5 levels under treatment (Bovibrom 225 ppm, Bovibrom 500 ppm, Fit Fresh 3 ppm, Washing Solution 750 ppm, Water). Pork loin section swabs at different storage times was a complete randomized design with repeated measures over time. All counts were analyzed using Kruskal–Wallis nonparametric test (R. Version 4.04), followed by pairwise multiple comparison Wilcoxon’s test adjusted by Benjamin & Hochber method. Wilcoxon’s test was used to identify the significant variation in microbial level on swab samples collected at different sampling points, storage times, and treatments. A *p*-value of 0.05 or less was selected prior to the analysis to determine significant differences in this study.

## 3. Results

### 3.1. Microbiological Analysis (before and after Treatment Application)

From pork loin sections, counts for coliforms, *Escherichia coli*, mesophilic aerobic bacteria (APC-M), psychrotrophic aerobic bacteria (APC-P), and mesophilic lactic acid bacteria (LAB-M) were performed before and after treatment application.

For all analysis, coliforms and *Escherichia coli* counts were below detection limit, <0.25 colony-forming unit (CFU)/cm^2^. Due to low initial counts, both coliforms and *Escherichia coli*, no statistical difference was found before and after treatment intervention. Low initial counts suggested that the plant from which samples were collected has implemented good manufacturing practices and dressing procedures and thus a good control of possible cross-contamination of endogenous sources of pathogens with pork carcasses.

**Figure 1:**
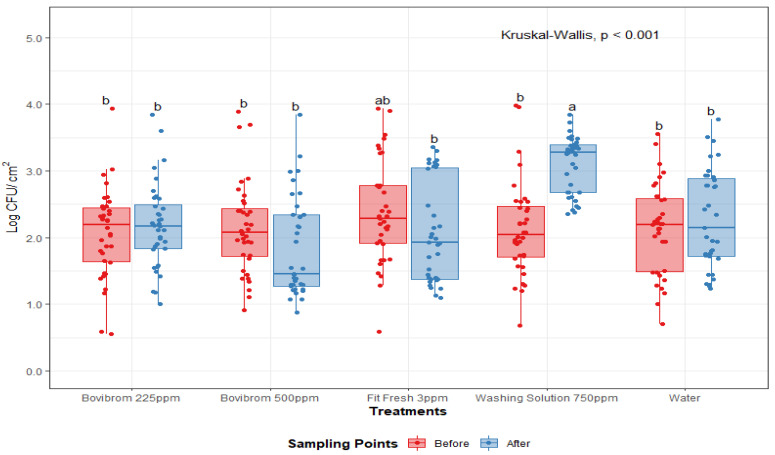
Mesophilic aerobic plate counts (Log CFU/cm^2^) before and after treatment application on pork loin sections (*n* = 36 per treatment). In each boxplot, the horizontal line crossing the box represents the median, the bottom and top of the box are the lower and upper quartiles, the vertical top line represents the upper interquartile range, and the vertical bottom line represents the lower interquartile range. Boxes with different letters a,b are significantly different according to Kruskal–Wallis analysis followed by pairwise comparison Wilcoxon’s test at *p* < 0.05. The points represent the actual data points. Active ingredients: Bovibrom = 1,3-Dibromo-5,5-dimenthylhydantoin; Fit Fresh = chlorine dioxide; Washing Solution = rhamnolipid.

**Figure 2:**
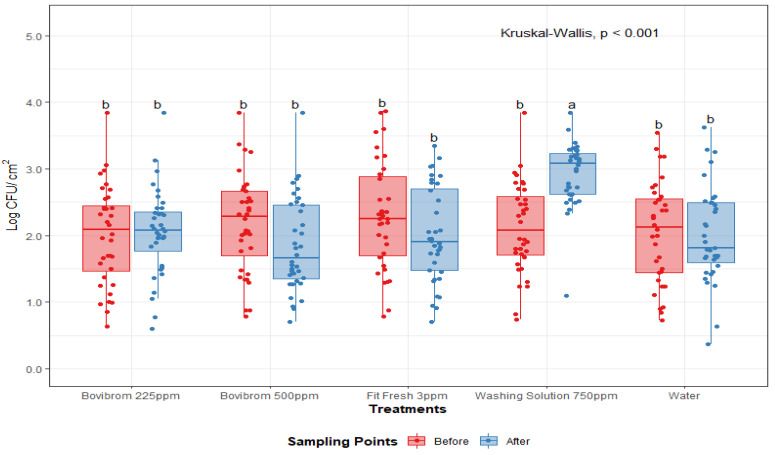
Psychrotrophic aerobic plate counts (Log CFU/cm^2^) before and after treatment application on pork loin sections (*n* = 36 per treatment). In each boxplot, the horizontal line crossing the box represents the median, the bottom and top of the box are the lower and upper quartiles, the vertical top line represents the upper interquartile range, and the vertical bottom line represents the lower interquartile range. Boxes with different letters a,b are significantly different according to Kruskal–Wallis analysis followed by pairwise comparison Wilcoxon’s test at *p* < 0.05. The points represent the actual data points. Active ingredients: Bovibrom = 1,3-Dibromo-5,5-dimenthylhydantoin; Fit Fresh = chlorine dioxide; Washing Solution = rhamnolipid.

A treatment by sampling point interaction was found for APC-M and APC-P (*p* < 0.01), (Figure 1 and Figure 2). For these microorganisms, initial counts (before intervention) did not differ between treatments, while after intervention, treatment with Washing Solution 750 ppm did not effectively reduced counts for APC-M and APC-P. For mesophilic lactic acid bacteria (LAB-M), no effect was found by treatment or sampling point (*p* = 0.69). After treatment application, counts for Washing Solution 750 ppm for APC-M and APC-P were statistically higher (*p* < 0.01), when compared to the other treatments, suggesting a lower antimicrobial efficiency immediately after intervention.

### 3.2. Microbiological Analysis (End of Dark Storage Time)

From pork loin sections, enumeration of APC-M, APC-P, coliforms, *Escherichia coli*, and LAB-M were performed at the end of each of four dark storage periods (1 Day, 14 Days, 28 Days, and 42 Days) at refrigerated temperatures 0–4 °C. Similarly, coliform and Escherichia coli initial counts were below detection limit (< 0.25 CFU/cm^2^) for the first day of storage. After 14 days, enumeration was above detection limit, and a statistical difference was found between counts at 14, 28, and 42 days (0.09, 0.52, 1.44 Log CFU/cm^2^, respectively; largest standard error: 0.09 Log CFU/cm^2^).

**Figure 3:**
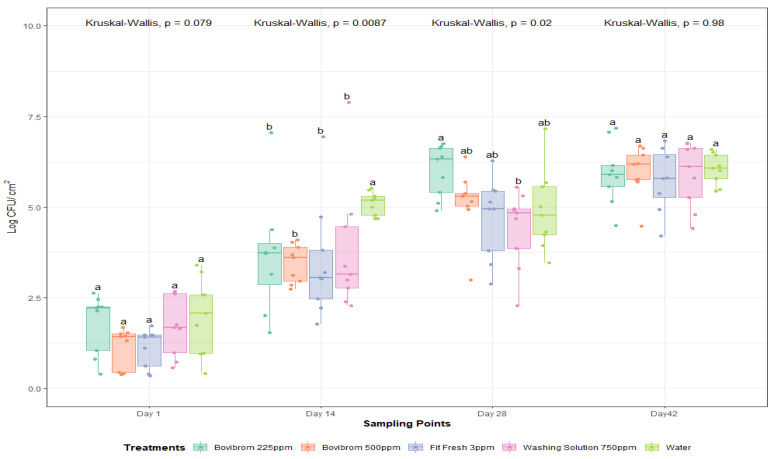
Mesophilic aerobic plate counts (Log CFU/cm^2^) after 1, 14, 28, and 42 days of dark storage time on pork loin sections (*n* = 45 per dark storage time). In each boxplot, the horizontal line crossing the box represents the median, the bottom and top of the box are the lower and upper quartiles, the vertical top line represents the upper interquartile range, and the vertical bottom line represents the lower interquartile range. For each sampling point day, boxes with different letters a,b are significantly different according to Kruskal–Wallis analysis followed by pairwise comparison Wilcoxon’s test at *p* < 0.05. The points represent the actual data points. Active ingredients: Bovibrom = 1,3-Dibromo-5,5-dimenthylhydantoin; Fit Fresh = chlorine dioxide; Washing Solution = rhamnolipid.

For APC-M, a dark storage time by treatment interaction was found (*p* = 0.05). As storage time was found to be significant, a statistical analysis was performed per storage time in order to find differences between treatments. Bacterial cells by nature multiply over time; that is why the biological importance lies in the change of treatments over time. Nonparametric approach tests, also called distribution-free tests, was used in order to analyze the results, because they do not assume that the data follow any specific distribution as parametric tests do. The distribution of the data (Figure 3) suggests neither samples follow a normal distribution, or the sample size was big enough. A Kruskal–Wallis test, the test used when assumptions of ANOVA are not met, was performed to find differences between treatments over the four dark storage times.

After one day of dark storage, counts were not different between treatments (*p* = 0.08). There were differences for Bovibrom 225 ppm, Bovibrom 500 ppm, Fit Fresh 3 ppm, and Washing Solution 750 ppm counts at 14 days of storage time when compared with Water (*p* < 0.01). Then, after 28 days of storage time, all treatments presented similar values when compared with Water. For all treatments, there was no immediate effect on the microbial load of pork loin sections (Day 1), but these results suggest that there was a residual effect of all the treatments by the fact that, at Day 14, all counts were lower when compared with Water. Moreover, these results show that this residual effect is lost by the time the pork samples reached 28 days of refrigerated storage time.

**Figure 4:**
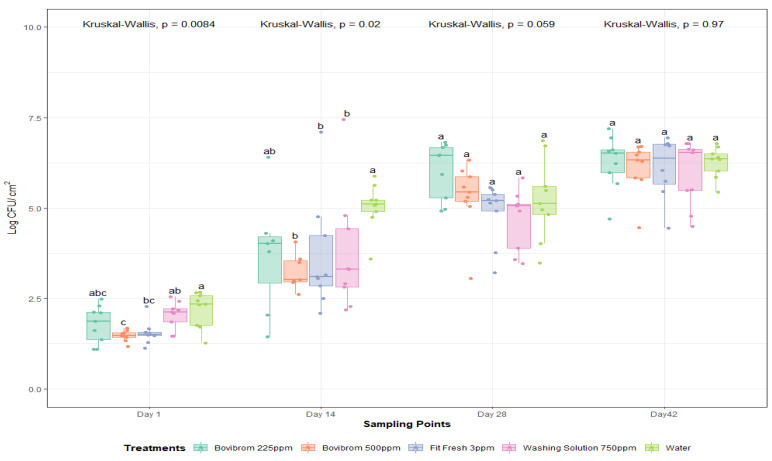
Psychrotrophic aerobic plate counts (Log CFU/cm^2^) after 1, 14, 28, and 42 days of dark storage time on pork loin sections (*n* = 45 per dark storage time). In each boxplot, the horizontal line crossing the box represents the median, the bottom and top of the box are the lower and upper quartiles, the vertical top line represents the upper interquartile range, and the vertical bottom line represents the lower interquartile range. For each sampling point day, boxes with different letters a,b are significantly different according to Kruskal–Wallis analysis followed by pairwise comparison Wilcoxon’s test at *p* < 0.05. The points represent the actual data points. Active ingredients: Bovibrom = 1,3-Dibromo-5,5-dimenthylhydantoin; Fit Fresh = chlorine dioxide; Washing Solution = rhamnolipid.

Further, for APC-P (Figure 4), a dark storage time by treatment interaction was found (*p* = 0.03). At Day 1 of dark storage, counts were not different between treatments. There were differences for Bovibrom 500 ppm, Fit Fresh 3 ppm, and Washing Solution 750 ppm counts at 14 days of storage time when compared with Water, but in later storage times, all treatments presented similar values compared with Water. Similar results were obtained for APC-M and APC-P related with the residual effect, found by using this type of interventions on pork loins. For mesophilic lactic acid bacteria (LAB-M), no significant effect was found for treatment or dark storage (*p* = 0.45).

## 4. Discussion

Each treatment has different active ingredients; therefore, the overall spectrum, the mode of action, and the efficacy against microorganisms are highly dependent on the chemical and physical properties of the antimicrobial [6]. Treatment with Washing Solution 750 ppm is a biosurfactant within the glycolipid category known as rhamnolipids, which are produced mainly by *Pseudomonas aeruginosa* [16,17]. Rhamnolipids is an amphipathic surface-active molecule composed of ß-hydroxy fatty acid connected to a rhamnose sugar molecule used for a broad range of applications, such as antimicrobial agents [18,19]. Its antimicrobial activity is related primary by damaging the cytoplasmic membrane, causing an increase in its permeability due to the release of lipopolysaccharides from the outer membrane [20,21,22].

In a study where rhamnolipids were tested at different concentrations for Gram-positive and Gram-negative bacteria, the antimicrobial effect was completely indistinguishable for Gram-negative bacteria at all concentrations tested, while Gram-positive bacteria were inhibited at most concentrations, explaining the lower antimicrobial efficiency when compared to other antimicrobials that have a larger overall spectrum of action [22,23].

In a pork chop shelflife study using organic (citric or ascorbic) acid applications and vacuum packaging system, psychrotrophic enumeration was performed in order to see the effect of these interventions in storage time up to 14 days. Results showed that, despite of the intervention and packaging system, psychrotrophic bacteria were still capable of growing over the storage period [24]; however, our study presented a clear decrease in log counts for APC-M and APC-P after 14 days of storage time when compared to the Water application. Evidence in this study suggests that antimicrobial interventions are effective during the 14-day period, but once the antimicrobial effects are depleted, the remaining bacteria have less competition to multiply, increasing the rate of growth up to the point that counts at 42 days of storage did not differ from water application.

Treatment Bovibrom (225 and 500 ppm) is a commercial name for the active ingredient 1,3-dibromo-5,5-dimenthylhydantoin (DBDMH) [25]. DBDMH is a bromanine polymer, which hydrolyzes to hypobromous acid (HOBr) in presence of water [26]. This hypobromous acid has the same biocide property as hypochlorous acid (HOCl), and they both combine with organic compounds to form bromanines or chloramine, respectively; however, bromanines are more potent than chloramines and, therefore, show more effectiveness in the presence of organic matter [6]. Halogen’s mechanism of action is not well defined, but theories such as interference in cell metabolism by oxidation of SH groups (sulfhydryl group) essential for bacterial enzymes due to pH or oxidation of purine and pyrimidine bases causing mutation are the most well-known and accepted [27]. Further, treatment with Fit Fresh 3 ppm is a commercial name for the active ingredient that in, presence of water, is known as chlorine dioxide. Chlorine dioxide differs from normal chlorine compounds, as it does not form HOCl, but it presents similarities in the antimicrobial activity due to the oxidation-reduction potential [6]. The mechanisms of action are not well defined, but it is theorized that protein synthesis and disruption of the outer membrane to be highly responsible for their antimicrobial activity [6].

Counts performed at the end of each dark storage time suggested that Fit Fresh, Bovibrom 500 ppm and Washing Solution 750 ppm performed better until 14 days of storage despite the wide spectrum of chlorine dioxide and hypobromous acid when compared with rhamnolipids. In addition, there is a clear increase in counts for all microorganisms as dark storage time increased, which is not surprising, because increased storage time in a vacuum bag will result in increased bacterial proliferation [28]. The use of antimicrobials is mainly to inhibit the growth of microorganisms by extending the lag phase of their lifecycle, and according to literature found, a meat product around 6 log CFU/cm^2^ is at a level in which it could be considered spoiled, even though microbial loads is not the only attribute to be considered for shelf life [29]. During the dark storage period, treatments with Bovibrom 500 ppm, Fit Fresh 3 ppm, and Washing Solution 750 ppm presented values below this limit, and it is not until 42 days of storage that the pork section approached this limit, suggesting that the increase of shelf life, considering microbiological characteristics using these antimicrobials, is accomplished. When compared to other studies where, after 28 days of aging this limit was reached, our study delayed reaching the 6-log population limit until 42 days of storage [30].

## 5. Conclusions

The purpose of the study was to determine the shelf life of pork loins with the application of different antimicrobials evaluating growth on common microbial indicators. The antimicrobials Bovibrom 500 ppm, Fit Fresh 3 ppm, and Washing Solution 750 ppm performed the best for maintaining reduced microbial counts when compared to Water in pork loins after 14 days of dark storage under refrigerated conditions 0–4 °C.

## Data Availability

The data presented in this study are available on request from the corresponding author.

## References

[1] NAMI (2016). Fact Sheet U.S. Meat and Poultry Production & Consumption: An Overview.

[2] USDA Per Capita Consumption of Pork in the United States from 2015 to 2028 (In Pounds). https://www.statista.com/statistics/183616/per-capita-consumption-of-pork-in-the-us-since-2000/.

[3] Baer A.A., Miller M.J., Dilger A.C. (2013). Pathogens of interest to the pork industry: A review of research on interventions to assure food safety. Compr. Rev. Food Sci. Food Saf..

[4] CDC (2018). Pathogens Causing the Most Illnesses, Hospitalizations, and Deaths Each Year.

[5] Institute of Food Science and Technology, Institute of Food Science and Technology (1993). Shelf-Life of Foods: Guidelines for Its Determination and Prediction.

[6] Davidson M., Sofos J.N., Branen A.L. (2005). Antimicrobials in Food.

[7] AOAC (2002). AOAC Official Method 990.12 Aerobic Plate Count in Foods.

[8] Dormedy E.S., Brashears M.M., Cutter C.N., Burson D.E. (2000). Validation of acid washes as critical control points in hazard analysis and critical control point systems. J. Food Prot..

[9] Goepfert J.M. (1976). The Aerobic Plate Count, Coliform and Escherichia coli Content of Raw Ground Beef at the Retail Level. J. Milk Food Technol..

[10] Jay J. (2002). A review of aerobic and psychrotrophic plate count procedures for fresh meat and poultry products. J. Food Prot..

[11] Ercolini D., Russo F., Nasi A., Ferranti P., Villani F. (2009). Mesophilic and psychrotrophic bacteria from meat and their spoilage potential in vitro and in beef. Appl. Environ. Microbiol..

[12] AOAC (2002). AOAC Official Method 991.14 Coliform and Escherichia coli Counts in Foods.

[13] Tortorello M.L. (2003). Indicator Organisms for Safety and Quality—Uses and Methods.pdf. J. AOAC Int..

[14] Moraes P.M., Perin L.M., Ortolani M.B.T., Yamazi A.K., Viçosa G.N., Nero L.A. (2010). Protocols for the isolation and detection of lactic acid bacteria with bacteriocinogenic potential. LWT Food Sci. Technol..

[15] Pothakos V., Devlieghere F., Villani F., Björkroth J., Ercolini D. (2015). Lactic acid bacteria and their controversial role in fresh meat spoilage. Meat Sci..

[16] Kaeppeli O., Guerra-Santos L. (1984). Process for the Production of Rhamnolipids. U.S. Patent.

[17] Randhawa K.K.S., Rahman P.K.S.M. (2014). Rhamnolipid biosurfactants-past, present, and future scenario of global market. Front. Microbiol..

[18] Xu Q., Nakajima M., Liu Z., Shiina T. (2011). Biosurfactants for microbubble preparation and application. Int. J. Mol. Sci..

[19] Magalhães L., Nitschke M. (2013). Antimicrobial activity of rhamnolipids against Listeria monocytogenes and their synergistic interaction with nisin. Food Control.

[20] Rahman P.K.S.M., Gakpe E. (2008). Production, characterization, and applications of biosurfactants. Biotechnology.

[21] Parry A., Parry N., Peilow C., Stevenson P. (2011). Combinations of Rhamnolipids and Enzymes for Improved Cleaning. WIPO Patent.

[22] Díaz De Rienzo M.A., Stevenson P., Marchant R., Banat I.M. (2016). Antibacterial properties of biosurfactants against selected Gram-positive and -negative bacteria. FEMS Microbiol. Lett..

[23] Devendra H., Venkata N., Smita S., Vayalam P. (2010). Rhamnolipid mediated disruption of marine Bacillus cereus biofilms. Colloids Surf. B Biointerfaces.

[24] Huang N.Y. (2005). Retail Shelf-Life of Pork Dipped in Organic Acid before Modified. J. Food Sci..

[25] Kalchayanand N., Arthur T., Bosilevac J., Schmidt J., Wang R., Shackelford S., Wheeler T. (2011). Efficacy of Commonly Used Antimicrobial Interventions on Shiga Toxin-Producing Escherichia coli Serotypes O45, O121, and Non-MDR and MDR Salmonella Inoculated Fresh Beef Final Report.

[26] Sun G., Allen L.C., Luckie E.P., Wheatley W.B., Worley S.D. (1995). Disinfection of Water by N-Halamine Biocidal Polymers. Ind. Eng. Chem. Res..

[27] Estrela C., Estrela C.R.A., Barbin E.L., Spanó J.C.E., Marchesan M.A., Pécora J.D. (2002). Mechanism of action of sodium hypochlorite. Braz. Dent. J..

[28] Hodges J.H., Cahill V.R., Ockerman H.W. (1974). Effect of Vacuum Packaging on Weight Loss, Microbial Growth and Palatability of Fresh Beef Wholesale Cuts. J. Food Sci..

[29] Banwart G.J. (1981). Basic Food Microbiology.

[30] Holmer S.F., McKeith R.O., Boler D.D., Dilger A.C., Eggert J.M., Petry D.B., McKeith F.K., Jones K.L., Killefer J. (2009). The effect of pH on shelf-life of pork during aging and simulated retail display. Meat Sci..

